# A Tutorial Review on the Fluorescent Probes as a Molecular Logic Circuit—Digital Comparator

**DOI:** 10.3390/molecules28176327

**Published:** 2023-08-29

**Authors:** Nikolai I. Georgiev, Ventsislav V. Bakov, Vladimir B. Bojinov

**Affiliations:** 1 Department of Organic Synthesis, University of Chemical Technology and Metallurgy, 8 Kliment Ohridsky Str., 1756 Sofia, Bulgaria; bakov@uctm.edu; 2 Bulgarian Academy of Sciences, 1040 Sofia, Bulgaria

**Keywords:** fluorescent probe, identity molecular digital comparator, magnitude molecular digital comparator, XNOR molecular logic gate, XOR molecular logic gate, INHIBIT molecular logic gate, IMPLICATION molecular logic gate

## Abstract

The rapid progress in the field of fluorescent probes and fluorescent sensing material extended this research area toward more complex molecular logic gates capable of carrying out a variety of sensing functions simultaneously. These molecules are able to calculate a composite result in which the analysis is not performed by a man but by the molecular device itself. Since the first report by de Silva of AND molecular logic gate, all possible logic gates have been achieved at the molecular level, and currently, utilization of more complicated molecular logic circuits is a major task in this field. Comparison between two digits is the simplest logic operation, which could be realized with the simplest logic circuit. That is why the right understanding of the applied principles during the implementation of molecular digital comparators could play a critical role in obtaining logic circuits that are more complicated. Herein, all possible ways for the construction of comparators on the molecular level were discussed, and recent achievements connected with these devices were presented.

## 1. Introduction

The great attention paid in the last thirty years to the design and fabrication of variety machinery and devices at the molecular level has led to significant progress, as a result of which this field has been expanded from intramolecular motion and molecular switches [1,2,3,4,5,6,7] to nanoscale motion in artificial muscles [8,9,10], nano cars [11,12,13], molecular cranes [14], molecular cursor caliper [15], molecular timers [16,17], molecular computing [18,19,20,21] and molecular cryptography [22,23].

**Figure 1:**
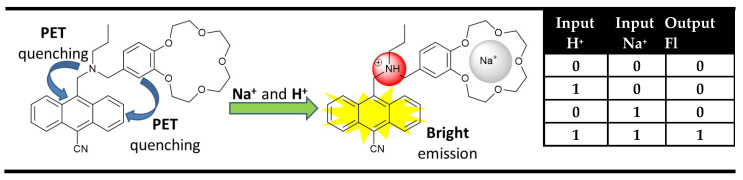
The first AND logic gate was reported by A.P. de Silva in 1993, and the corresponding truth table [27].

One of the most important developments in the last time was made in semiconductor-based computer science and technology. Currently, computers perform binary arithmetic and logical operations using semiconductor logic gates as elementary units building digital circuits. The logic operations are based on the Boolean algebra in which the FALSE (NO) and TRUE (YES) are encoded in binary code as “0” and “1”, respectively. Usually, the semiconductor logic gates operate via two inputs and one output, which states they possess one of the two binary conditions “0” or “1” encoded using a low (0) or high (1) voltage. This process is achievable on the molecule level in many ways, but the most common ones are based on the optical switching properties of molecule chemosensors [24,25], using low and high analyte concentrations as inputs, resulting in an “off” or “on” state of the optical output. The idea for the molecular-logic-gates-based computation, initially started by Aviram’s pioneering work as an alternative minimization of the semiconductor logic gates, actually is a major factor for fast computation in conventional computers (miniaturizing electronic components to a very small size results in an electron travel on very short distances in a very short time) [26]. However, the new era of molecular Boolean logic operations began five years later after the work by de Silva, who illustrated that the ability of fluorescent probes to deal with binary “1” and “0” inputs allows the fabrication of logic gates using fluorescence as an output [27]. The proposed AND logic gate represented a PET (Photoinduced Electron Transfer) based molecular chemosensor containing two different selective receptor fragments—tertiary amine for H^+^ (input 1) and benzo crown ether moiety for Na^+^ (input 2). In order to obtain a strong fluorescence signaling output from the anthracene fluorophore, the PET quenching processes needed to be suppressed from both receptors. In the presence of protons, the PET from the alkylamine receptor was interrupted, but fluorescence emission was not observed because of the PET quenching effect from the crown ether. The presence of sodium cations inhibits a PET process from the crown ether but not from the amino receptor, and the system again does not fluoresce. Simultaneous use of both H^+^ and Na^+^ interrupts both PET processes to the anthracene fluorophore, resulting in strong fluorescence. In binary, the resulting behavior satisfied AND logic gates (Figure 1).

Since the first report by de Silva et al. [27], the field of molecular logic gates has advanced considerably. All possible logic gates were achieved at the molecular level, and a series of synthetic molecules and biomolecules have been utilized in the molecular computation, including proteins, enzymes, DNA, and RNA [28,29,30,31,32,33,34,35,36]. In addition, examples of more complicated logic circuits performing arithmetic functions [37,38], simple games such as Tic-Tac-Toe [39,40], and molecular scale anticancer terminator automaton [41,42] were reported. The illustrated logic gates have found practical applications in object coding [43], sensing systems [44,45,46,47], data storage [48,49], and drug delivery and drug activation systems [50,51,52,53]. All these molecules are able to calculate a composite result autonomously and often were determined as labs on the molecularly level. Furthermore, molecular computation could be applied to intelligent medical diagnostics where abnormal results are detected and interpreted using Boolean logic algebra during a medical examination. Such molecular devices can save time for doctors and are crucial when health services are in extreme conditions, for example, in epidemics or bioterrorism [54]. Therefore, despite the significant results achieved in this field, the construction of complex logic circuits with practical applications is still a great challenge. Due to the different nature of the used chemical inputs and optical outputs, the consecutive integration of molecular logic gates in molecular circuits is seriously restricted. Generally, the consecutive integration of fluorescent probes in logic circuits is achieved using multichromophoric systems and FRET communication between the separated gates [55]. The parallel integration of molecular logic gates is usually achieved by monitoring the different changes in the absorption and fluorescence spectra at different wavelengths at the same time. Currently, the reported molecular logic circuits use more “virtual” than physical integration of individual logic gates. The signaling output at various channels and thresholds (fluorescence and/or absorption at different wavelengths) are typically analyzed, and then a suitable set of logic gates is proposed to generate an apparent logical circuit that is only limited by the researcher’s creativity. This virtually gives more flexibility for the creation of logic circuits; therefore, some molecules are able to perform the action of more than one logic circuit [38,56].

Comparison between two digits is the simplest logic operation, which could be realized with the simplest logic circuit. The right understanding of the applied principles during the implementation of molecular digital comparators could play a critical role in obtaining logic circuits that are more complicated. However, review papers in the literature are very rare. Generally, the reported reviews were focused on the design and fabrication of molecular logic circuits such as devices for addition and subtraction, key lock pads, multiplexers, demultiplexers, encoders, decoders, “write-read-erase-read” function [16,19,20,24,25,57,58,59,60,61]. Even the monograph by de Silva [28], which is an essential inspiration in this field, is a detailed look at digital comparators, and they were only briefly reported. Therefore, in this tutorial review, all possible ways for the construction of comparators on the molecular level are discussed, and the recent achievements connected with these devices are presented. First, the XNOR and XOR molecular logic gates are introduced to the reader as the simplest logic device for comparison called the identity comparator. Then, more complicated molecular logic circuits operating as magnitude comparators are presented using two or three parallel acting gates in positive or negative logic. We believe that the presented information will be in help for researchers who wanted to be familiar with the molecular logic gates and molecular logic circuits.

## 2. Identity Molecular Digital Comparators (XNOR and XOR Molecular Logic Gates)

**Figure 2:**
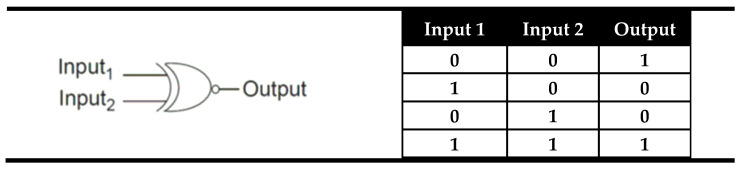
The truth table and electronic representation of the XNOR logic gate.

In general, there exist two basic types of digital comparators: identity comparators and magnitude comparators [62]. The simplest logic device able to make a comparison between two binary digits is an XNOR logic gate whose output is “1” (TRUE) only when both inputs are equal. The truth table and the electronic representation of an XNOR logic gate are presented in Figure 2. As can be seen from Figure 2, the XNOR can say that both inputs are equal, but if they are not equal, it cannot say which one is greater or less than other. This type of comparators that indicate only whether two inputs are equal or not equal were named Identity comparators. The digital comparators that signify which input is larger or smaller, if they are not equal, were called magnitude digital comparators and they will be presented later.

**Figure 3:**
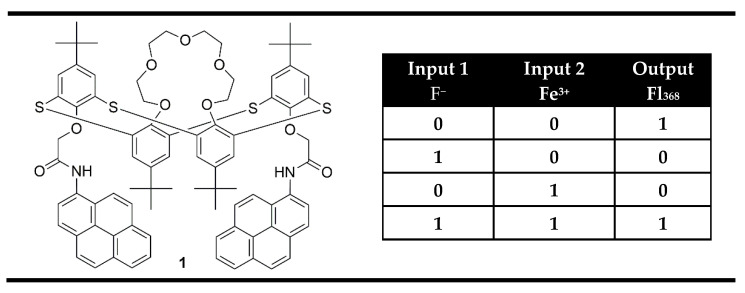
XNOR molecular logic gate based on fluorescent properties of probe **1** and corresponding truth table [63].

The easiest way to obtain XNOR gates on the molecular level is based on the fluorescent probes that bind two fluorescent quenching analytes, which are able to interact with each other and so together to annihilate their individual effects. Compound **1** (Figure 3) was presented as an example of such a system [63]. It was designed as a pyrene appended probe using thiacalix[4]arene of 1,3-alternate conformation as a highly selective receptor towards Fe^3+^ and amide NH for selective interaction with F^−^ anions. In the absence of both analytes, the probe showed a strong fluorescence emission at 386 nm. The presence of Fe^3+^ ions induced fluorescence quenching due to the paramagnetic nature of Fe^3+^ and reverse PET from pyrene units to the nitrogen atom. In addition, toward fluoride ions, compound **1** gave another fluorescence quenching response that was attributed to the hydrogen bonding of the amide moiety, leading to the formation of a static excimer. The addition of fluoride ions to a solution of **1**-Fe^3+^ complex recovers the probe **1** fluorescence emission. The fluorescence revival process upon the addition of F^−^ ions indicates that Fe^3+^ has more affinity for F^−^ ions than with the amide NH receptor of probe **1**. The observed fluorescence changes coded in binary as output upon the actions of two chemical inputs are correlated very well with the XNOR logic gate (Figure 3).

**Figure 4:**
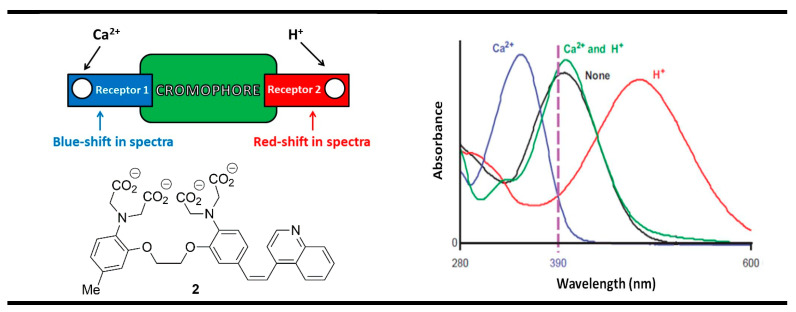
XNOR molecular logic gate based on fluorescent properties of probe **2**. Adapted from *J. Am. Chem. Soc.* **2000**, *122*, 3965–3966 [64] with permission from the American Chemical Society.

Another useful concept for the rational designing of XNOR molecular logic gates was proposed by de Silva and McClenaghan using receptor_1_-chromophore-receptor_2_ ICT (Internal Charge Transfer) chemosensing architecture [64]. The main peculiarity of this model was laid on the opposite response of both analyte-bounded receptors. The binding process in the first receptor should result in a blue spectral shift, while in the second receptor, the shift is in the direction of longer wavelengths. This was very well illustrated with the ICT probe **2** for the detection of biologically important cations—Ca^2+^ and H^+^ (Figure 4).

In the excited state of compound **2**, a dipole arises due to an internal charge transfer (ICT) process from the electron-rich tertiary aniline receptor to the electron-accepting quinoline moiety in the molecule of the probe. The binding of the Ca^2+^ ion by the aniline moiety strongly reduced its electron density, thus destabilizing the ICT state of the compound and producing a blue shift in the absorption spectrum (Figure 4). At the same time, the protonation of the quinoline nitrogen receptor in the system resulted in a red shift owing to the increased electron-accepting ability of the protonated quinoline that increased the ICT efficiency of the system. The monitoring absorbance at 390 nm decreased in each case after the addition of Ca^2+^ and protons as chemical inputs because the absorption band moved away from the monitoring wavelength. The simultaneous action of both ionic inputs virtually cancels each other due to the opposite influences, and the absorption spectrum of compound **2** practically was returned to its initial state. Therefore, in binary, we have an XNOR logic gate, where a “high” output is registered only when both inputs are equal.

**Figure 5:**
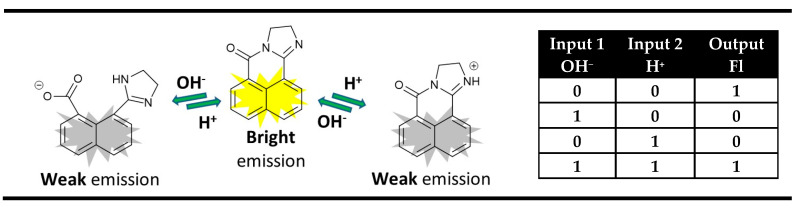
XNOR molecular logic gate based on fluorescent properties of the dihydroimidazo 1,8-naphthalimide derivatives and corresponding truth table [65,66,67].

The ingenious strategy of acid-base neutralization as a means of achieving the required cancellation of inputs in “off-on-off” pH-sensing fluorescence probes is an excellent platform for the implementation of an XNOR logic gate on the molecular level (Figure 5).

A typical example of that is the dihydroimidazo 1,8-naphthalimide derivatives that showed strong fluorescence at neutral pH. The addition of acid or base quenched the fluorescence signal due to the protonation or decyclization of the dihydroimidazo unit [65,66,67]. It was found that this process is reversible, and the neutralization of the acid and base regenerates the strong fluorescence of the probes. Hence, the monitoring of fluorescence emission in these derivatives as an output using acid and base as chemical inputs results in an XNOR logic gate at the molecular level (Figure 5).

**Figure 6:**
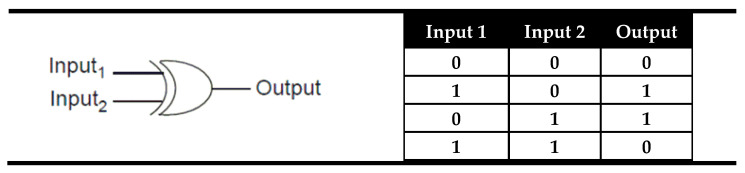
The truth table and electronic representation of the XOR logic gate.

XOR is the other logic gate that could be used as an identity comparator. This gate is logically connected with the XNOR logic gate using negative logic. The negative logic is the logic in the “land of liars” where the TRUE is presented as FALSE and FALSE as TRUE. In other words, negative logic is convention for the “high” output signals as logic state “0” and “low” signals as logic state “1”. The convention between normal and negative logic is called logic inversion and is sometimes invoked in semiconductor technology. In digital logic, the NOT gate is used as an inverter that implements logical negation owing to the opposite output state compared to the input. The truth table and the electronic representation of the XNOR logic gate are presented in Figure 2. As can be seen from Figure 6, in the XOR gate, the signal output is coded as “0” only when both inputs are equal and is coded as “1” if the inputs are different. This behavior is exactly the opposite of the XNOR gate, and here “0” is a sign of the input equality instead of “1” (Figure 6).

**Figure 7:**
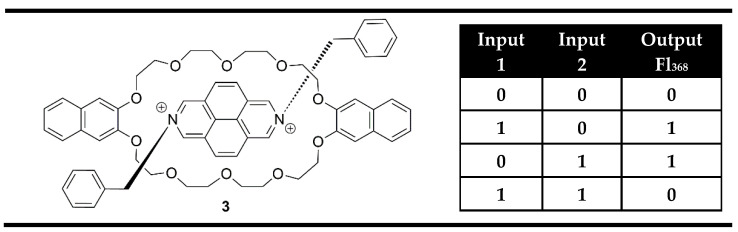
XOR molecular logic gate based on the formation of pseudorotaxane **3** and corresponding truth table [68].

Among all logic gates, the XOR logic gate is the most difficult to implement at the molecular level. The first example of an XOR logic gate at the molecular scale was demonstrated by Balzani, Stoddart et al. [68] using the pseudorotaxane **3** (Figure 7), which was obtained after self-assembly of the electron-accepting 2,7-dibenzyldiazapyrenium dication (Input 1) with the crown ether containing two 2,3-dioxynaphthalene units (Input 2). Separately, both pseudorotaxane components exhibited bright fluorescence. Due to the electron donor/acceptor interaction in compound **3**, a low energy charge transfer excited state is formed, and the strong former fluorescence of the inputs disappears. The monitoring of fluorescence as output and the individual pseudorotaxane moieties as inputs during the formation of pseudorotaxane **3** satisfies an XOR logic gate (Figure 7). However, it should be pointed out that the observed logic gate is not based on a molecule but on a volume of solution in the lack or presence of the chemical inputs.

Now, the most popular approach for the fabrication of XOR logic gates at the molecular level is based on the negative logic of XNOR gates operating via absorption outputs [64,69,70,71,72,73]. The observed XNOR gates in the absorption mode could easily be transformed in an XOR logic gate by logical inversion of the output by monitoring transmittance instead of absorption as output. The absorbance and transmittance are inversely related. Thus, if a “high” transmittance output was demonstrated, a “low” output via an absorbance would be obtained. Usually, the logical inversion by monitoring of the transmittance or negative logic was used in order to obtain XOR, which is required for the construction of a half-adder, a logical device for the addition of two binary digits. In fact, the above-mentioned XNOR molecular gate **2** [64] was reported by de Silva as transmittance of the XOR achieved gate in this context.

**Figure 8:**
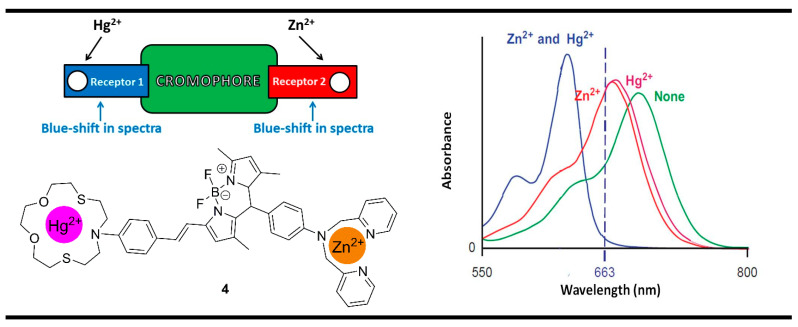
XOR molecular logic gate based on fluorescent properties of probe **4**. Adapted from *J. Am. Chem. Soc.* **2010**, *132*, 8029–8036 [74] with permission from the American Chemical Society.

One of the rarest examples of XOR molecular logic gates using positive logic of the chemosensing probes was reported by Akkaya [74] with the BODIPY system **4** for the recognition of Zn^2+^ and Hg^2+^ (Figure 8). Probe **4** is an ICT-based sensing architecture configured on the receptor_1_-chromophore-receptor_2_ format. For a difference of the above-mentioned model proposed by de Silva and McClenaghan for the construction of the XNOR gate (Figure 4), here the analyte-bounded receptors showed a very similar blue-shifting response, which plays a key role in obtaining the desired XOR gate. Due to the strong ICT nature of compound **4**, it showed an absorption spectrum with a maximum of 679 nm. When Zn^2+^ ions were added, the blue shifting to 674 nm was observed. A similar shift to 673 nm takes place after the addition of Hg^2+^. The resulting small individual effects of the electron-accepting analytes were explained with partially blocked ICT process after binding with the electron-rich amino-containing receptor fragments. However, when both ions were added the ICT state in system **4** was strongly destabilized due to the accumulated influence of the cations and the absorption of the new formed metal-bound complex was shifted further toward the shorter wavelengths at 630 nm. That is why the recoding of absorbance at 663 nm, where only the presence of each analyte alone showed a high absorption output, gives an XOR gate in binary.

**Figure 9:**
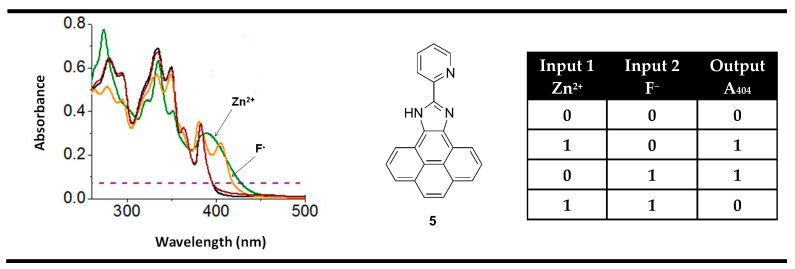
XOR molecular logic gate based on fluorescent properties of probe **5** and corresponding truth table. Adapted from *Sens. Actuators B: Chem.* **2015**, *206*, 701–713 [75] with permission from Elsevier.

An interesting example of an XOR molecular logic gate using positive logic was presented by Mardanya et al. probe **5** [75]. The pyridyl-imidazole-based chromophoric system in compound **5** showed good chemosensing properties toward ions of Zn^2+^ and F^−^. Similarly to XOR gate **4**, the recognition of any one of the analytes resulted in the appearance of the absorption bands at the same place—around 404 nm. Thus, the monitoring of absorption spectra at 404 nm of compound **5** in binary allows the implementation of an XOR logic gate at the molecular level (Figure 9).

## 3. Magnitude Molecular Digital Comparators

**Figure 10:**
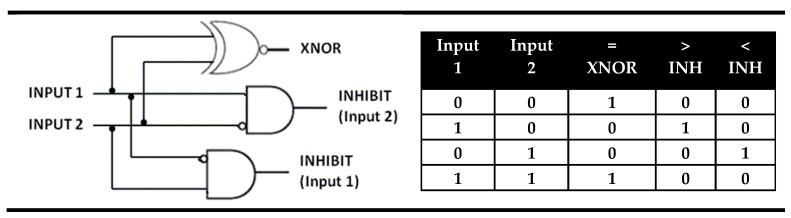
Truth table and electronic representation of magnitude digital comparator.

The magnitude digital comparators are logical devices that perform full comparison between two binary digits. A rational analysis of such a device reveals that it should be constructed on an architecture comprising two inputs and three parallel outputs. One of the outputs should have the value TRUE or binary “1” only when the inputs are equal and will be a sign of the inputs’ equality (“=”). Other output should be TRUE (binary “1”) only when the first input alone has a high value (coded as “1”), which will be a sign that the first input is greater (“>”) than the second. Additionally, the third output should show TRUE (binary “1”) as a signaling behavior only when the value of the second input is “1” in order to point out that the first input is less than (“<”) the second one. The parallel performance of these three outputs in a logic circuit satisfied all requirements for comparison of two binary digits hence it fulfills the action of the magnitude digital comparators. In the magnitude digital comparator the above discussed XOR gate is the used output for equality while two INHIBIT logic gates give the outputs “greater than” and “less than”. In fact, the magnitude digital comparator is identity comparator that is parallel extended with two INHIBIT logic gates. The INHIBIT gates output is high or “1”, only at a high value of the first or the second input. This logic gate was called INHIBIT because one of the inputs does nothing else than cancel the action of another. There are two possible INHIBIT gates: INHIBIT (Input 1) and INHIBIT (Input 2). In the INHIBIT (Input 2), which serves as the output “greater than” in the magnitude comparator, the second input disables the gates output. In contrast in INHIBIT (Input 1) the first input is the disabler which allows its function as the “less than” output in magnitude comparator. The truth table and electronic representation of magnitude digital comparators are presented on Figure 10.

**Figure 11:**
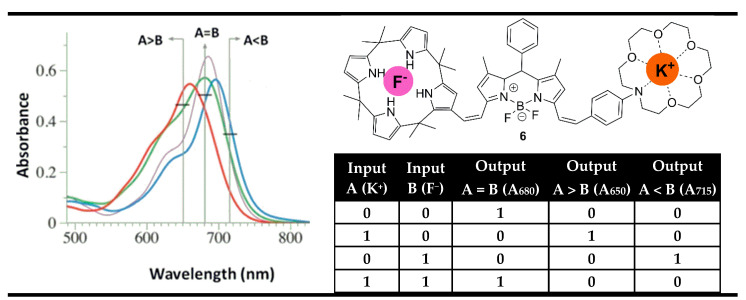
Molecular magnitude digital comparator based on absorption properties of probe **6** and corresponding truth table. Adapted from *Chem. Commun.* **2013**, *49*, 11056–11058 [79] with permission from the Royal Society of Chemistry.

In contrast to their semiconductor cousins, molecular logic devices are more flexible and can perform the work of several logic gates at the same time by monitoring the different changes in the absorption and fluorescence spectra at different wavelengths [28,76,77,78]. Furthermore, this flexibility results in a parallel action of several gates, allowing the construction of parallel logic circuits on a single molecule. This feature of the parallel measurements at different wavelengths is the base for the fabrication of digital magnitude comparators at the molecular level using optical outputs. For instance, Rurack et al. reported a multi-readout molecular gate capable of comparing K^+^ and F^−^ as binary inputs using BODIPY dye **6** (Figure 11) with alkali metal ion and anion binding sites [79].

The binding of a positively charged K^+^ ion at the crown unit leads to hypsochromic (blue-shifted) absorption due to the weakened ICT process as a result of reduced electron-donor strength of the bonded aniline nitrogen atom. On the other hand, the binding of a negatively charged F^−^ anion at the calix[4]pyrrole in compound **6** leads to a bathochromic (red) shift. The presence of K^+^ and F^−^ reduced their opposite individual effects and returned the absorption spectrum of **6** near the former state. Hence, the recording of the absorption spectrum of probe **6** at shorter wavelengths (650 nm) showed high binary output only in the presence of K^+^ as an input alone, which is well correlated with the INHIBIT logic gate. In addition, the monitoring at a long wavelength (715 nm) gives another IHIBIT gate in which only F^−^ is able to generate high absorption output. Meanwhile, the desired molecular comparator XNOR logic gate was achievable by monitoring the initial maximal absorption at 680 nm. As can be seen from Figure 11, the resulting parallel-acting logic gates completely cover the truth table of the digital magnitude comparator. The absorption output at 650 nm “greater than” is set to “1” (TRUE) when the value of input K^+^ is higher than F^−^. If the K^+^ input is less than F^−^ then the output at 715 nm named “less than” is set to “1”. Finally, if the comparison between the both inputs gives equality the output “equal” at 680 nm is set to “1”.

In molecular logic architectures, the parallel action of three or more rationally selected logic gates is a real challenge. Fortunately, the magnitude digital comparator could be obtained using two gates instead of three. This is possible due to the specific connection between the three outputs, which always have a high value on one of them and a low value on the two others —after all, a comparison of two digits could give only one of the outputs “equal”, “greater than”, or “less than” at the same time. This connection allows the achievement of magnitude comparison only using two gates because if the output of two of the gates is low, then the third one necessarily must be high, and its measurement is not required. The one explained here was the most exploited logic in the design of magnitude comparators at the molecular level, and the majority of the reported systems operate on the parallel action of XNOR and one INHIBIT gate [37,62,78,80].

**Figure 12:**
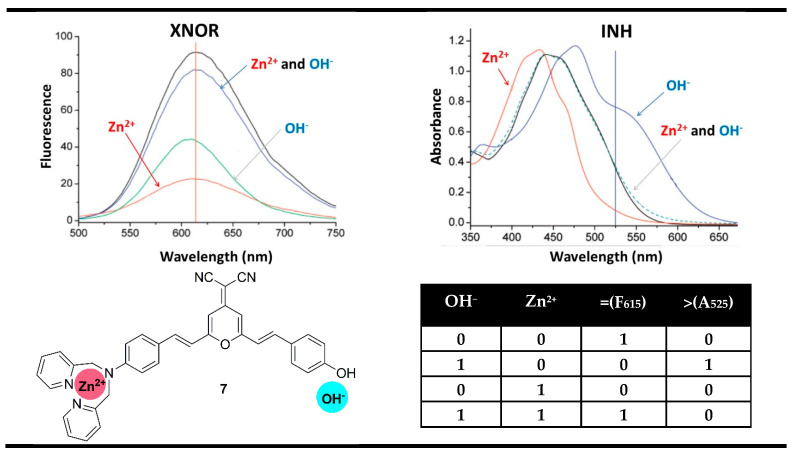
Molecular magnitude digital comparator based on absorption properties of probe **7** and corresponding truth table. Adapted from *J. Phys. Chem. C* **2008**, *112*, 7047–7053 [62] with permission from the American Chemical Society.

For instance, the reported by Tian et al. probe **7** for selective recognition of Zn^2+^ cations and OH^−^ anions (detection of Zn^2+^ in alkaline media) could serve as a magnitude digital comparator in this manner [62]. In the absence of Zn^2+^ and OH^−^ ions, compound **7** showed an absorption band centered at 442 nm and fluorescent emission at 615 nm. The addition of OH^−^ deprotonated the phenolic electron-donating fragment in probe **7** and increased its electron-donating ability. Thus, the ICT efficiency increased, and the absorption band of **7** was red-shifted to 484 nm, which was accompanied by a strong fluorescence quenching. The complexation of Zn^2+^ with compound **7** bound its amino substituent, thus destabilizing the exited ICT process, which resulted in blue shifting to 415 nm and additional fluorescence quenching. The presence of Zn^2+^ and OH^−^ simultaneously returned the absorption spectrum of probe **7** to the starting site and turned on the fluorescence emission at 615 nm. That is why the monitoring of the fluorescence at 615 nm in Boolean logic resulted in an XNOR gate. At the same time, based on the changes at 525 nm, where a high output was observed only in the presence of OH^−^, an INIHIBIT gate was constructed. The authors have used these two logic gates to construct a magnitude comparator at a molecular level. If both inputs are equal, then the fluorescence output at 615 nm is TRUE (“1”). If the OH^−^ input is greater than Zn^2+^, then the absorbance output at 525 nm is TRUE (“1”). When both outputs are FALSE (“0”), then the input OH^−^ is less than input Zn^2+^ (Figure 12).

**Figure 13:**
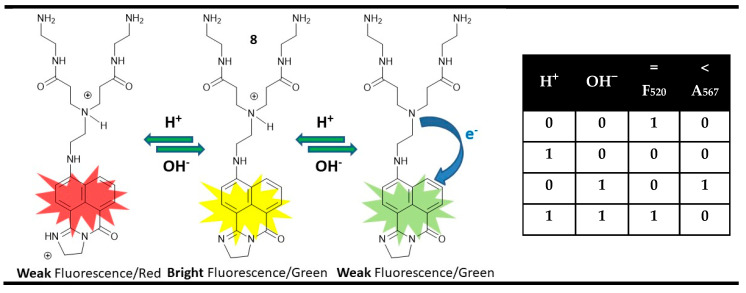
Molecular magnitude digital comparator based on fluorescent and absorption properties of probe **8** and corresponding truth table [81].

The simplest approach to obtain magnitude comparators at a molecular level is based on the annihilating nature of the acids and bases as inputs in the “off-on-off” pH chemosensing probes possessing two easily distinguished “off” states. In this model, the “off-on-off” signaling is used to construct a molecular XNOR gate starting from “on” state that could be recovered after the canceled actions of the two inputs in their simultaneous work. The distinction of both “off” states is required for the implementation of the wanted additional INHIBIT gate. A typical example is the PET-based dihydroimidazo 1,8-naphthalimide derivative **8** [81]. This molecule exhibits bright green fluorescence emission at 520 nm in neutral pH, where it is in a monoprotonated form due to the protonation of the tertiary amino PET receptor. In the presence of OH^−^ as an input, due to the possible PET quenching process in neutral form, compound **8** shows low fluorescence output at 520 nm. After the addition of acid to probe **8**, fluorescence quenching accompanied by bathochromic (red) shifting of the absorption spectrum from 510 nm to 567 nm is observed, which is due to protonation and increasing electron accepting ability of imido-imino substituent that enhanced the ICT nature of excited compound **8**. This results in an XNOR logic gate at the molecular level using the fluorescence changes at 520 nm that could be used as equal output during comparison. Additionally, it was found that the addition of acid that provokes a high output at 567 nm generates an IHIBIT logic gate at a molecular level that could be canceled in the presence of OH^−^. This indicates that the H^+^ input is high than OH^−^ output. Thus, the “off-on-off” fluorescence output of compound **8** possessing different absorption spectra of the both “off” states could be applied as digital magnitude comparator (Figure 13). Moreover, compound **8** is water-soluble and low toxic, so it could be a very helpful tool in clinical diagnostics because it compares pH levels to the normal physiological pH.

**Figure 14:**
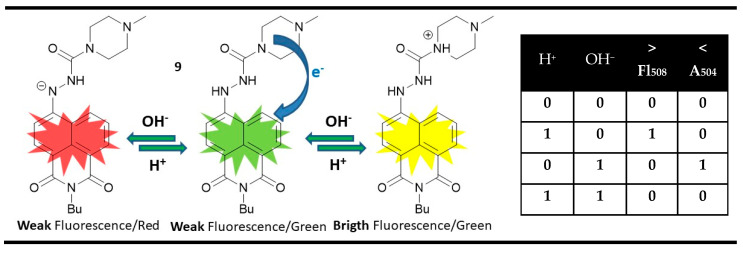
Molecular magnitude digital comparator based on fluorescent and absorption properties of probe **9** and corresponding truth table [82].

A magnitude digital comparator could be implemented at a molecular level using parallel performing INIHIBIT gates instead of one XOR and one INHIBIT gate. A rare example of that is 1,8-naphthalimide **9**, which is a dual-channel pH-responsive probe [82]. The yellow-green emitting compound **9** showed only weak fluorescence due to the PET quenching from the piperazine amines. When adding acid as an input, the PET was blocked, and a bright emission at 508 nm was observed as a fluorescence output. The presence of OH^−^ as a chemical input resulted in a non-emissive red-colored state with maximal absorption at 504 nm due to the enhanced ICT in deprotonated 4-hydrazino-1,8-naphthalimide chromophore. Based on these changes and the disabling action between the acid and hydroxide as outputs, parallel INHIBIT (input 1) and INHIBIT (input 2) gates could be constructed. Furthermore, the parallel performance of INHIBIT (input 1) and INHIBIT (input 2) in compound **9** allows digital comparison at a molecular level. When the H^+^ input is greater than OH^−^ one, the fluorescence output is high. If the H^+^ input is less than OH^−^ one, the absorption output is high. The low signaling values of both outputs indicate the equality of both inputs (Figure 14).

**Figure 15:**
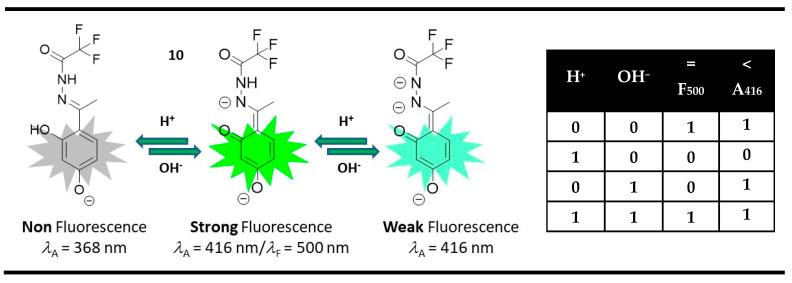
Molecular magnitude digital comparator based on fluorescent and absorption properties of probe **10** and corresponding truth table [83].

The use of negative logic could be helpful during the construction of a molecular magnitude comparator. For instance, compound **10** performs a comparison of two digital inputs, H^+^ and OH^−^, using XNOR and IMPLICATION gates [83]. The IMPLICATION logic gate is related to the INHIBIT gate by logic inversion (negation). Contrary to INHIBIT logic gates, whose output is high only at a high value of the one input alone, the IMPLICATION gate shows a low output signal in this case and high at the other three combinations. Thus, the IMPLICATION logic gate could replace the INHIBIT gate during comparison. At pH 11, compound **10** is in its fluorescent dianion form, which was quenched in the presence of acid and base as outputs. The addition of acid formed a non-fluorescent form due to a possible ESIPT process, while the addition of base quenched the emission due to the deprotonation of the amide fragment in probe **10**. Furthermore, for a difference of the OH^−^ effect, the H^+^ shifted the absorption of compound **10** from 416 nm to 368 nm. The annihilating nature of the used acid/base inputs and the monitoring of the absorption and fluorescent changes gives parallel-acting XNOR and IMPLICATION logic gates, whose activity allows comparison at the molecular level. If both inputs are equal (H^+^ = OH^−^), then both outputs are high. If the input H^+^ is greater than OH^−^ one, both outputs are low. If the input H^+^ is less than OH^−^ one, then the fluorescence output is low, but the absorption output is high (Figure 15).

**Figure 16:**
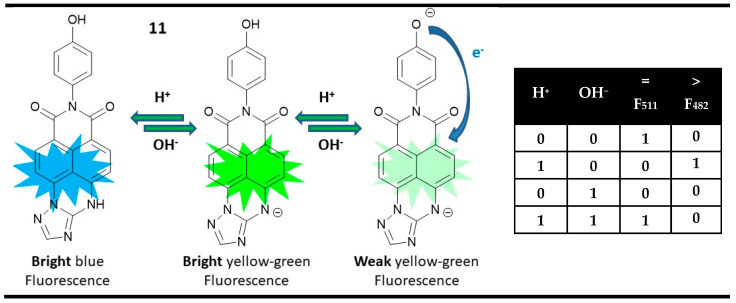
Molecular magnitude digital comparator based on fluorescent and absorption properties of probe **11** and corresponding truth table [84].

Probe **11** is an interesting example of a molecular magnitude comparator, which works only in the fluorescent mode [84]. As a mono anion, compound **11** showed a strong yellow-green fluorescence at 511 nm. After the addition of acid as an input, due to the formation of a neutral molecule with reduced ICT efficiency, the fluorescence was blue-shifted to 482 nm. On the other hand, the addition of OH^−^ resulted in a non-emissive dianion due to the presence of a PET quenching process from the formed phenolate anion. The presence of both acid and base cancels each other out, and the system returns to the starting yellow-green fluorescent form. The parallel monitoring of the fluorescence intensities at 511 nm and 482 nm results in obtaining XNOR and INHIBIT logic gates, respectively, whose performance is the above-mentioned classical requirement for the construction of a magnitude digital comparator at a molecular level, where the XNOR gate is “equal” output, while INHIBIT is “greater than”. Furthermore, the use of a paramagnetic quencher such as Cu^2+^ ion as a third chemical input disables both fluorescence outputs. Hence, based on probe **11,** a magnitude digital comparator with disabled capability at a molecular level could be achieved (Figure 16). The enable/disable capability could play an important role in blocking the unnecessary and setting up the required gates during the configuration of multicomponent systems, which now is a serious drawback at the molecular level.

## 4. Conclusions

In this tutorial review, all possible ways for the construction of digital comparators at the molecular level are discussed, and the recent achievements connected with these devices are presented. Comparison between two digits is the simplest arithmetic operation, and it could be realized with the simplest logic circuit. We hope the presented information will help young scientists to understand this field better and join it. The progress of molecular logic gates in the last thirty years is already impressive, but there are still plenty of outlets for the creative and innovative development of new concepts and applications for molecular computing of fluorescence probes. Improved recycling reset capabilities, reconfigurability, and integration of molecular logic gates on solid support will undoubtedly be an important theme. The higher-order logic circuits that could perform a comprehensive analysis of more than three targets are difficult to design at the molecular level. That is why such circuits need to be better designed and conceived. In addition, further intensive work on the functional integration of multichromophoric systems and interconnected cascades in supramolecular assemblies of multiple logic devices will be a great challenge.

Further extension of the principles of logic to applications in biomolecular recognition or theranostics will be the ultimate goal. While there has been success in the direction of logic operations, computational algorithms, sensing, and communication, some of the more unsolved fundamental challenges refer to concepts of intracellular applications. This is an area that ‘‘silicon-based computing cannot reach’’ and an important direction that needs to be studied more deeply in the future. Some fluorescent probes are related to the pathophysiological conditions in specific diseases; thus, a strategy could be designed of molecular logic based on this relationship that could complete the intelligent analysis of multi-targets, giving a unique YES or NO output. For many diseases, it is difficult to obtain an accurate diagnosis using a single or limited number of fluorescence probes, and it is necessary to conduct a comprehensive analysis of different permutations and combinations of multiple targets. Fortunately, the molecular logic gates enable the highly sensitive detection of multiple types of targets and the production of multiple signaling outputs. This development of molecular and computer science makes it a promising tool for clinical use. Although complex molecular logic gates are still not readily available in the clinic, and they serve only as proof of concept, healthcare professionals could greatly benefit from their existence.

As a whole, this exciting research area offers a great future working field that is limited only by the researcher’s imagination and inspiration.

## Data Availability

The authors declare that the data supporting the findings of this study are available within the article.
